# The ASPREE Healthy Ageing Biobank: Methodology and participant characteristics

**DOI:** 10.1371/journal.pone.0294743

**Published:** 2024-02-29

**Authors:** Emily J. Parker, Suzanne G. Orchard, Tom J. Gilbert, James J. Phung, Alice J. Owen, Trevor Lockett, Mark R. Nelson, Christopher M. Reid, Andrew M. Tonkin, Walter P. Abhayaratna, Peter Gibbs, John J. McNeil, Robyn L. Woods

**Affiliations:** 1 School of Public Health and Preventive Medicine, Monash University, Melbourne, Victoria, Australia; 2 Health and Biosecurity, Commonwealth Scientific and Industrial Research Organisation, North Ryde, New South Wales, Australia; 3 Technical Director, Rhythm Biosciences Ltd, Parkville, Victoria, Australia; 4 Menzies Institute for Medical Research, University of Tasmania, Hobart, Tasmania, Australia; 5 ANU Medical School, Australian National University, Garran, Australian Capital Territory, Australia; 6 Department of Medicine, The University of Melbourne, Melbourne, Victoria, Australia; Colorado State University, UNITED STATES

## Abstract

ASPirin in Reducing Events in the Elderly (ASPREE), a placebo-controlled prevention trial of low dose aspirin, provided the opportunity to establish a biospecimen biobank from initially healthy persons aged 70+ years for future research. The ASPREE Healthy Ageing Biobank (ASPREE Biobank) collected, processed and stored blood and urine samples at -80degC or under nitrogen vapour at two timepoints, three years apart, from a willing subset of Australian ASPREE participants. Written informed consent included separate opt-in questions for biomarker and genetic testing. Fractionated blood and urine were aliquoted into multiple low-volume, barcoded cryotubes for frozen storage within 4 hours of collection. Specially designed and outfitted mobile laboratories provided opportunities for participation by people in regional and rural areas. Detailed, high quality demographic, physiological and clinical data were collected annually through the ASPREE trial. 12,219 participants contributed blood/urine at the first timepoint, 10,617 of these older adults provided 3-year follow-up samples, and an additional 1,712 provided saliva for DNA. The mean participant age was 74 years, 54% were female and 46% lived outside major cities. Despite geographical and logistical challenges, nearly 100% of blood/urine specimens were processed and frozen within 4 hours of collection into >1.4 million aliquots. After a median of 4.7 years, major clinical events among ASPREE Biobank participants included 332 with dementia, 613 with cardiovascular disease events, 1259 with cancer, 357 with major bleeds and 615 had died. The ASPREE Biobank houses and curates a large number of biospecimens collected prior to the clinical manifestations of major disease, and 3-year follow-up samples, all linked to high quality, extensive phenotypic information. This provides the opportunity to identify or validate diagnostic, prognostic and predictive biomarkers, and potentially study biological effectors, of ageing-related diseases or maintenance of older-age good health.

## Introduction

Biobanks are recognised as an integral component of modern biomarker discovery and validation. A growing number of biobanks collect biospecimens coupled with associated clinical data for use in areas such as biomarker discovery for disease prognosis and tracking, drug discovery, and genome/phenome linkage. Many biobanks are disease-specific [e.g., 1, 2] and seek to support discovery of new therapeutic targets for a particular disease. Often, these biobanks are hospital based, collecting samples from patients after diagnosis or during follow-up. For convenience, most contributions to biobanks are from city-based participants. Biobanks that are not focussed on a single disease are more flexible as they can support a variety of studies, including cross-sectional studies of genotype-phenotype correlations, nested case control studies using a biobank for cases and/or controls, and cohort studies using baseline biobank and follow-up data that can link biomarker or genetic variation with health outcomes [3, 4]. There are a growing number of population-based biobanks collecting biospecimens and data from community-based cohorts of people [e.g., 5, 6] with the UK Biobank as a notable example, collecting biospecimens from more than 500,000 people aged 40–69 [www.ukbiobank.ac.uk; e.g, 7].

The ASPREE (ASPirin in reducing Events in the Elderly) clinical trial (refs) provided a unique opportunity to establish the ASPREE Healthy Ageing Biobank (hereafter ASPREE Biobank) from an initially healthy, older population, in a cost-effective manner by utilising the clinical trial infrastructure to enrol older participants. Throughout the trial, high quality phenotypic data were collected annually from participants in person, and identification of major clinical events was supported by collation of medical documentation and adjudication by panels of experts. The continued engagement of participants in the trial provided the opportunity for a second set of blood and urine specimens added to the ASPREE Biobank in order to track changes in biomarkers over time and to allow future investigations into biological mechanisms underpinning aspirin-related effects on health outcomes. With the collection of biospecimens linked to the ASPREE clinical trial data the ASPREE Biobank provides a valuable resource for future research into diseases of ageing and factors associated with healthy ageing. This paper describes the Biobank methods, numbers of participants, types and numbers of samples in the collection, the profile of the cohort and clinical outcomes of interest to researchers. The benefits of employing a mobile laboratory to access volunteers residing outside of major metropolitan areas are considered.

## Methods

### ASPREE features

ASPREE was a multi-centre, randomised, double-blind, placebo-controlled trial of daily 100mg enteric-coated aspirin in 19,114 healthy community dwelling older adults in Australia (n = 16,703) and the U.S. (n = 2,411). By the end of the clinical trial, ASPREE had determined that daily 100mg aspirin over a period of 4.7 years did not extend disability-free or dementia-free survival in older adults [8]. The ASPREE study methods have been described in detail elsewhere [9]. Briefly, community-based recruitment occurred 2010–2014, with participants followed up annually in person until the trial was ended in 2017 [8, 10, 11]. Data captured during this period included demographics, cognitive and physical function, mood, lifestyle (e.g., alcohol intake, smoking and living status), quality of life and medication use. Participants’ clinical events were tracked longitudinally and study endpoints, including dementia, cancer, myocardial infarction, stroke, hospitalisation for heart failure, clinically significant bleeding and cause of death, were adjudicated and confirmed by expert panels provided with de-identified clinical source documents and masked to the randomisation group [9]. Study endpoints of depression and persistent physical disability (loss of independence with any basic activity of daily living) were evaluated through annual participant questionnaires. The pre-specified outcome of frailty was assessed, and hospitalisations were collected and coded. Death was confirmed by two independent sources and cause of death (trajectory) was adjudicated from clinical records. Exclusion criteria for the ASPREE study included a past history of a cardiovascular event or established cardiovascular disease, dementia, independence-limiting physical disability, a condition with a high current or recurrent risk of bleeding, anaemia, a condition likely to cause death within 5 years, current use of other antiplatelet or antithrombotic medication, current use of aspirin for secondary prevention, and uncontrolled hypertension (≥180/105mmHg).

After the completion of the intervention phase of the ASPREE trial, all participants were invited to continue in an observational extension cohort called ASPREE-eXTension or ASPREE-XT. More than 14,000 Australian ASPREE participants consented to continue in ASPREE-XT, with annual study measures and clinical outcomes collected as described above for ASPREE, providing on-going longitudinal follow-up of their health and well-being.

### Biobank recruitment and consent

All ASPREE participants in Australia were eligible to provide samples for the ASPREE Biobank within 12 months of randomisation to trial drug (100mg aspirin or placebo). Workflows were constructed to prioritise biospecimen collection to within the first 4 weeks of enrolment and, where possible, prior to initiation of study medication. Participants provided opt-in written consent for their samples to be used for biomarker and/or genetic analysis. The ASPREE Biobank operates under ethics approval number 18/08 provided by the Alfred Hospital Ethics Committee, Melbourne, as the primary ethics site; regional ethics committees provided approval through University of Tasmania, ACT Health, University of Adelaide.

Biobank participants were recruited through ASPREE study visits. Each participant received the participant information and consent form at the initial baseline ASPREE study visit, allowing the opportunity to thoroughly read and discuss the sub-study with their GP or family members. At the second ASPREE baseline visit, after which participants were randomised to the clinical trial medication if they met all criteria for the trial, participants could have any queries about biobank participation answered by study staff. The provision of informed consent by ASPREE participants to contribute to the Biobank sub-study included agreement to the future use of their biospecimens in unspecified medical research studies (subject to such studies having ethics committee approval), that all samples would be de-identified (barcoded only) for storage, and that their samples would not be sold.

**Fig 1:**
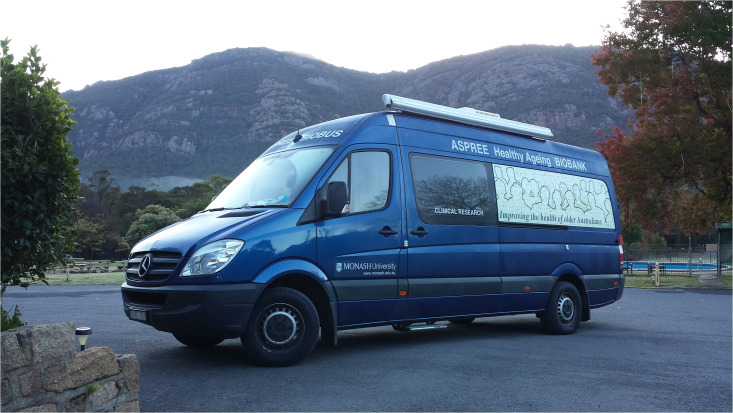
Mobile laboratory used to access regional and rural/remote participants to collect and process biospecimens. Legend: Vehicle outfitted with laboratory grade benches, refrigerated centrifuge, refrigerator, disposable equipment in secure cupboards and drawers, air conditioning, a bed readily converted to an additional bench, a chair for participants, electric step for easy access, a -80 degC portable cryoshuttle and/or LN2 resin insert cryoshipper, an external powerpoint and an external generator to provide power when not connected to main power.

ASPREE Biobank ‘baseline’ samples were collected 2010–2015, predominantly in parallel with the ASPREE clinical trial recruitment period; follow-on samples for each participant were collected ~3 years after the initial sample. Strategies were developed to enable biospecimen collection close to where participants lived including in rural/regional or remote areas whilst complying with study protocols. These strategies included setting up regional processing laboratories, developing customised mobile laboratories (Fig 1) and utilising commercial pathology networks in metropolitan and regional centres. The ASPREE ‘Biobus’ mobile laboratories were custom designed, enabling trained staff to collect, process blood and urine samples, and store them on site at -80°C in regional areas where other laboratory facilities were unavailable. If required, contracted pathology service centres located close to the participant’s home were engaged to collect the blood and one of our staff couriers provided the ice and timely transport of bloods to a processing laboratory or the Biobus.

### ASPREE Biobank sample collection

All ASPREE Biobank/biorepository procedures are based on quality standards approved by the International Society for Biological and Environmental Repositories (ISBER; www.isber.org). A decentralised approach to biospecimen handling enabled collection, processing and storage of frozen samples within 4 hours at each site before shipping in bulk, on dry ice, to the central biorepository for long-term storage. Details of each collection (and processing procedures, see below) were documented onto a Biospecimen Information Sheet, which tracked collection details such as time of collection, time of specimen arrival at the laboratory, processing duration, whether the participant had commenced ASPREE trial medication (100 mg aspirin or placebo), the time of the last meal prior to sample collection, whether any open-label aspirin was taken in the previous week and whether the participant had ever had a blood transfusion and if so, the date of the transfusion (S1 Table).

Approximately 40 mL of non-fasting blood, collected into 5 blood collection tubes consisting of 3 ethylene diaminetetraacetic acid (EDTA; 10mL capacity) tubes, 2 sodium citrate (NaC; 2.7mL capacity) tubes and 1 serum separation transport (SST; 8.5mL capacity, Serum BD Vacutainer^™^) tube, and up to 20 mL of urine were required for a full collection of each participant’s biospecimens. Sub-division into storage aliquots involved a maximum of 28 EDTA-plasmas, 10 NaC plasmas, 10 serum aliquots, 6 buffy coat aliquots, 4 packed red blood cell aliquots, 6 urine aliquots and one 9-spot Guthrie card. Aliquots were stored in multiple 0.5 mL 2D-barcoded cryovials, housed within a one-D barcoded ‘plate’. Samples were maintained on ice (or at 4degC) during all steps of processing and fractions separated in refrigerated centrifuges. Blood samples were collected by ASPREE Biobank staff or phlebotomists at pathology providers by prior arrangement. Samples were processed as soon as practicable to maintain the 4-hour window, with initial storage at -20degC or -80degC, depending on availability at the facility.

Saliva was collected into Oragene-DNA kits (http://www.dnagenotek.com/ US/products/OGR250.html) from consented participants who were unable or unwilling to provide a blood sample. These saliva kits are designed for the collection of human DNA from saliva samples and contain stabilisation buffers suitable for storage at room temperature. Sample collections occurred at the ASPREE study visit unless it was deemed more practical to have the participant complete the saliva sampling at home and return by mail. Collections were conducted at least 30mins after eating (including chewing gum), drinking or smoking, and a full collection (up to 2 mL) was completed within 30 mins, with all of these details tracked on a Biospecimen Information Sheet. On receipt of the completed saliva sample kit at the central ASPREE Biobank laboratory, the saliva sample was catalogued (via unique barcode) and stored at room temperature with no further processing required until the time of DNA extraction.

### ASPREE Biobank sample processing

Prior to centrifugation, nine blood spots (each 60 μL) from one EDTA blood tube were added to a Guthrie card (Whatman filter paper, Grade CF12) for each participant. Vacutainers were then centrifuged at 4degC and the upper layers of plasma from each EDTA collection tubes were pooled into a single use 15 ml collection tube and the approximate volume recorded on a Biospecimen Information Sheet (S1 Table). The maximum number of aliquots, in 2D barcoded NUNC^™^ cryovial tubes (ThermoFisher Scientific), per participant was 64 with the default aliquot volume of 250 or 500 μL. When the volume of blood collected was less than maximum, 250 μL aliquots were stored to maintain the aliquot number at 64. A Sample Aliquot Volume Guide was devised providing a quick reference tool for the person processing samples to ascertain the number of 500 μL and 250 μL aliquots required to complete the full tube set number without leaving any left over. The mid white blood cell layer (buffy coat) between the EDTA plasma and red cell matter was carefully removed, with as little contamination as possible from the layers above and below, and then twice washed in red blood cell lysis buffer (pH = 7.3) and re-suspended in phosphate-buffered saline (PBS) before being aliquoted into 6 cryovials (250 μL each). Packed red blood cells (RBC) were collected from the remaining EDTA blood tube, 500 μL aliquoted into each of 4 cryovials. Plasma from the NaC vacutainers was also pooled into a single use 15 ml tube, and the approximate volume recorded. The Sample Aliquot Volume Guide was used to estimate the maximum volume (distributed between either 250 μL or 500 μL) required to generate a set of 10 NaC cryovials. The blood sample processing description above was followed for all baseline collections. For the year 3 blood collections, buffy coat samples were aliquoted directly and not washed in lysis buffer and four buffy coat aliquots were stored rather than six.

Participants provided up to 70ml of urine in a sterile urine collection jar at the Biobank visit. For pragmatic reasons, allowing for collection at any time during the day, there were no requirements for this sample to be from the first urination of the day, mid-stream, or from a fasting sample. Along with other details collected about the samples (detailed below under the section on Storage and Curation), the time since the last food intake was recorded. Urine was stored at 4degC or on ice until distributed into six 500 μL aliquots in 2D barcoded NUNC tubes at baseline and four 500 μL aliquots at year 3.

A full collection of each participants’ biospecimens included non-fasting blood (~40 mL) collected into 6 blood tubes (3 EDTA tubes, 2 NaC tubes and 1 serum tube) and ~20 mL of urine. A full set of stored aliquots after fractionation and processing comprised 28 EDTA-plasmas, 10 NaC plasmas, 10 sera, 6 buffy coats, 4 packed RBC, 6 urines and one 9-spot Guthrie card. Aliquots were stored in 0.5 mL 2D-barcoded NUNC^™^ cryovials (ThermoFisher Scientific), housed within a one-D bar coded plate with default volumes of 250 or 500 μL for most aliquots. Plasma samples were maintained on ice (or at 4degC) during all steps of processing and fractions separated in refrigerated centrifuges. Samples were processed as soon as practicable to maintain the 4-hour window, with initial storage at -20degC or -80degC, followed by long term storage at -80degC or in LN2 vapour tanks. Total plasma or serum volumes were recorded.

### Storage and curation

Once frozen at -80°C for short-term storage, cryovials of serum and two thirds of the plasma samples were subsequently transferred to nitrogen vapour phase tanks, whilst aliquots of buffy coat, packed RBC, the remaining plasma and urine were retained at -80°C for long-term storage. Each participant’s sample collection set was scanned via a flatbed scanner (NUNC 2D Barcode Scanner and Bioscan 96 Software) that linked the 2-D barcode to the participant identifier number and acrostic. This linking of sample to participant was entered into a laboratory information management system (LIMS—Nautilus; Thermo Scientific) along with relevant sample details collected on the Biospecimen Information Sheet. Processing details were also captured on this form: the number and types of aliquots stored, the time and type of each storage step, and comments indicating any factors which may impact the sample, including but not limited to, the presence of haemolysis, any thaw events, collection tube malfunctions or any other anomalous factors observed during collection, processing or storage that may impact on sample quality. Data entry into the LIMS was de-identified but linked to the participant through the ID code and acrostic, and cross checked by an independent second person check. Only approved, trained biobank staff who conducted or processed the collected samples, and who signed confidentiality agreements, have access to the LIMS or participant IDs through secure password protected log-in. Subsequent re-arraying of aliquots provided back-up samples to be housed in a separate location for safety of the collection and to maximise storage space. Each aliquot was traceable through the 2D barcode linked to its location on the plate, to the plate’s location in the tower or shelf, to the tower/shelf location in the freezer, and to the freezer’s location in the room and/or building. All stored samples were identified only through the 2D-barcode etched into the storage cryovial, with no other personal information linked to the tubes. Investigators and analysts do not have access to personal information that could identify individual participants during or after biomarker or data analysis.

### Staff training, monitoring and quality control

Laboratory staff were trained to conduct the sample processing according to detailed standard operating procedures (SOP), with regular monitoring of adherence to collection and processing protocols at each location, and other aspects of biobanking activity including consenting and phlebotomy. To maintain sample quality, regular staff monitoring was performed to ensure collection and processing protocols were adhered to, including at regional sites. Prior to establishing a processing site for ASPREE Biobank samples, the laboratory was site checked for facilities and the ability of staff to follow good laboratory practice. If required, Biobank staff from Melbourne travelled to a regional site periodically to collect and process samples within a suitable local hospital or University laboratory location. The meta-data collected at the time of biospecimen collection and processing (e.g., times and temperatures) were reviewed regularly for adherence to the SOPs. In the event that an independent site was not functioning to satisfaction, the laboratory was re-trained in the Biobank SOPs and if there were still issues, the laboratory was no longer used for ASPREE biobanking processing.

### Governance and sample access policy

Access to ASPREE Biobank biospecimens requires approval through the ASPREE Governance committee and independent ethics approval. Considerations include: (i) the potential scientific value of the project; (ii) experience and quality of the researcher(s) and laboratory conducting the analyses; (iii) numbers, type and quantity of specimens requested; (iv) any overlap with existing projects; (v) any ethical considerations related to return of results and (vi) funding availability for the project including sample retrieval and shipping. The ASPREE governance process includes a two-step request where access to the biospecimen is reviewed by the ASPREE Biospecimen Access Committee and access to accompanying de-identified participant data is reviewed by the ASPREE Principal Investigators. All requests for access are through the ASPREE Access Management System (www.ams.aspree.org). Prior to the provision of samples for approved projects, samples are re-coded with a new project-specific participant ID and only the Biobank Manager and a member of the ASPREE Data Management Team hold the key to this newly coded list.

## Results

### Recruitment statistics

Of the 16,703 Australian participants in the ASPREE trial, 14,113 (84%) consented to provide samples for biobanking, whilst 2,024 (12%) declined any level of Biobank participation. The remaining 4% of participants neither consented nor formally declined. Of those who consented, 12,219 (86.6%) subsequently provided blood and/or urine samples or Guthrie card. Reasons for consented participants not providing samples were mainly logistical and related to timing between consent (at the ASPREE study visit) and participant availability at the time when convenient collection options were available (e.g., access to sample collection location or a Biobus). Rarely, no blood or urine could be collected due to changed participant preference or difficulty with phlebotomy or urine voiding. Of those who did not provide a blood sample for whatever reason, 1,712 consented to and provided a saliva sample for germline DNA. Eight participants (<0.07%) indicated they did not want their blood samples analysed for genetics.

In total, 66% (8,113) of blood/urine samples were collected prior to commencement of aspirin or placebo and an additional 14% (1,700) were collected within one month of commencement of allocated study medication. Nearly all samples were collected within a year of randomisation to ASPREE with a mean time for collection after randomisation of 50.2 ± 84.7 days. Of those approached to provide a year 3 follow-up sample, 10,617 provided follow-up blood and/or urine for biobanking. The mean (SD) time between ASPREE randomisation date and year 3 sample collection was 3.24 (0.37) years.

### Collection locations

**Fig 2:**
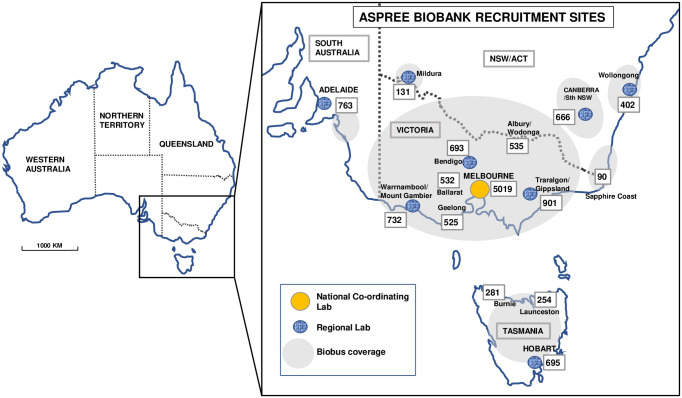
Recruitment map of ASPREE Biobank participants. Legend: Map of Australia with rectangle of enlarged south-eastern area showing the major ASPREE Biobank recruitment sites (named) and include the number of Biobank participants recruited from each site. Processing laboratories are indicated by the dotted oval symbols, as are the regions covered by the mobile biobuses indicated by shaded oval areas. The percentages of ASPREE participants recruited to Biobank in each location, relative to the total number of ASPREE participants in each site (listed alphabetically), were Adelaide, SA (61%), Ballarat, VIC (75%), Bendigo, VIC (79%), Burnie, TAS (42%), Canberra, ACT/Southern NSW (66%), Geelong, VIC (73%), Hobart, TAS (78%), Launceston, TAS (46%), Melbourne, VIC (78%), Mildura, VIC (75%), Traralgon, VIC (77%), Warrnambool VIC/Mt Gambier, SA (83%), Wodonga/Albury, VIC/NSW (72%), Wollongong, NSW (76%). A regional site established in Shepparton, VIC closed after collecting 15 samples.

ASPREE Biobank samples were collected from all 16 Australian ASPREE recruitment sites [9]. These were concentrated in south-eastern Australia and included sites in Victoria, Tasmania, South Australia, New South Wales and the Australian Capital Territory (Fig 2). The proportion of randomised ASPREE participants per site who contributed blood/urine to the Biobank ranged from 42% (Burnie, Tasmania) to 83% (Warrnambool, Victoria), with a mean of 73% across all sites. Lower percentages were in locations where participants were randomised to ASPREE earlier in the recruitment period, prior to establishing a biobanking service in the area. During the eight years of Biobank collections, three contract laboratories in regional locations were disengaged for biobanking, due to lack of adherence to the SOPs.

### Stored samples

#### Baseline samples

Of the baseline collections, 74.8% had a complete set of 64 aliquots, whilst 92.5% consisted of 60+ aliquots (Table 1). The average collection was 62 tubes per participant set. Guthrie cards (9 blood spots of 60 μL volume) were prepared for 12,027 (98.4%) of Biobank participants. EDTA plasma and SST serum form the majority of stored blood aliquots (Table 1). A small proportion (1.8%) of baseline samples contained only urine aliquots when blood was not able to be collected. Nearly 760,000 aliquots of blood components or urine aliquots were stored from baseline samples (Table 1).

**Table 1:** Sample types and numbers stored at baseline and at year 3.

***(a) BASELINE TIMEPOINT***								
		**500 μL aliquots**		**250 μL aliquots**		**All aliquots**		
**Sample Type**	**No. Pts**	**Total**	**Av/pt**	**Total**	**Av/pt**	**Total**	**Av/pt**	**Target /pt**
EDTA Plasma	11,985	225,095	18	108,390	9	333,485	27	28
NaC Plasma	11,820	11,137	1	103,415	8	114,542	9	10
SST Serum	12,011	43,341	4	75,600	6	118,931	10	10
RBC	11,980	47,881	4	-	-	47,881	4	4
Urine	12,021	71,807	6	-	-	71,807	6	6
Buffy coat	11,985	71,815	6	-	-	71,815	6	6
**Total**	**12,219**	**471,076**	**39**	**287,405**	**23**	**758,481**	**62**	**64**
***(b) YEAR 3 TIMEPOINT***								
		**500 μL aliquots**		**250 μL aliquots**		**All aliquots**		
**Sample Type**	**No. Pts**	**Total**	**Av/pt**	**Total**	**Av/pt**	**Total**	**Av/pt**	**Target /pt**
EDTA Plasma	10,371	194,086	18	96,099	9	290,185	27	28
NaC Plasma	10,230	6,727	1	91,657	9	98,384	9	10
SST Serum	10,373	37,516	4	65,632	6	103,148	10	10
RBC	10,356	41,628	4	-	-	41,628	4	4
Urine	10,225	61,340	6	-	-	61,340	6	6
Buffy coat	10,368	41,732	4	-	-	41,732	4	4
**Total**	**10,617**	**341,297**	**37**	**295,120**	**24**	**636,417**	**60**	**62**

This table shows the number of participants with at least 1 aliquot of each sample type; Av/pt columns display the average number of aliquots of each sample type, to the nearest whole number, across the entire Biobank cohort of participants with stored blood and/or urine at (a) baseline (12,219) or (b) year 3 (10,617). Target/pt is the maximum number of tube types per participant’s collection. Total No. Pts is the number of participants with blood or urine components stored. EDTA = ethylenediaminetetraacetic acid, NaC = sodium citrate, SST = serum separator tube, RBC = packed red blood cells.

#### Year 3 samples

Eighty seven percent (10,617) of the ASPREE participants who donated samples at baseline, also provided a second blood and/or urine samples 3 years later. Overall, 65.4% of year 3 collections were complete with a full set of 62-aliquots whilst 90.3% consisted of 58 or more aliquots (Table 1). A small proportion (1.3%) of year 3 samples contained only urine aliquots. Nearly all year 3 blood collections, similar to baseline, had at least one of each aliquot stored (Table 1).

### Timing of sample processing procedures

Only 78 samples (0.6%) failed to meet the 4-hour target of collection-to-freezer for the baseline sample collection. Sample transport and processing times are illustrated in S1 Fig. Almost three quarters of all samples (74%) reached the processing laboratory within 1 hour of collection, and 79% were processed within 2 hours. The mean (SD) times for sample transport and sample processing of baseline samples were 44 (33) and 103 (27) minutes, respectively. The majority of samples (73%) had a total time from collection to storage of between 90 and 180 minutes, with a mean of 148 (39) minutes. Data for collection, transport, processing and storage times were similar for the year 3 samples (data not shown). Regardless of processing conditions, all samples remain in the Biobank collection with meta-data about conditions and timing for each sample accessible to investigators to make their own decision as to whether or not to include the samples for analysis.

### Demographics

The demographic characteristics of the Biobank cohort closely matched those of the entire Australian ASPREE participant cohort [9] in terms of age distribution, gender, education levels, ethnicity and rurality (Table 2). Approximately 60% of Biobank participants were aged between 70–74 years, whilst 40% were 75+ years with 3% aged 85+ years. The median age of Biobank participants at baseline sample collection was 74.1 (IQR 5.7) years and the oldest participant was 96 years; 54% were female.

**Table 2:** Demographics of ASPREE Biobank participants.

	Biobank participants (N = 12,219)	AUS ASPREE participants* (N = 16,703)
**Age (yrs)**		
70–74	7,314 (60%)	9,668 (58%)
75–79	3,176 (26%)	4,432 (26%)
80–84	1,342 (11%)	1,963 (12%)
85+	387 (3%)	640 (4%)
**Gender**		
Men	5,672 (46%)	7,523 (45%)
Women	6,547 (54%)	9,180 (55%)
**Education (yrs)**		
<12	5,984 (49%)	8,399 (50%)
≥12	6,235 (51%)	8,303 (50%)
**Ethnicity / race**		
White	11,967 (98%)	16,362 (98%)
African American	4 (0.0%)	4 (0.0%)
Hispanic	91 (0.7%)	115 (0.7%)
Asian	86 (0.7%)	128 (0.7%)
Other	71 (0.6%)	94 (0.6%)
**Rurality**		
Major Cities of Australia	6,584 (53.9%)	8,730 (52.3%)
Inner Regional Australia	4,382 (35.9%)	5,984 (35.8%)
Outer Regional Australia	1,224 (10.0%)	1,951 (11.7%)
Remote Australia	0 (0.0%)	3 (0.0%)
Not available	29 (0.2%)	35 (0.2%)

Baseline characteristics data are for those who contributed blood and/or urine to Biobank. Headers show the numbers (N) of participants in the Biobank sub-study and main ASPREE study* [9]. Row values show numbers and % of total. The category of “White” includes those who did not identify as Hispanic and identified as White/Caucasian. Rurality categories are defined by the Australian Bureau of Statistics

Almost 98% of participants identified as white Caucasian whilst 2.1% identified as another ethnicity, including African American, Hispanic, Asian, Aboriginal/Torres Strait Islander, Native Hawaiian/Pacific Islander or more than one race. Overall, 54% of Biobank participants lived in major cities of Australia whilst 36% were living in inner regional areas and 10% were in outer regional or remote areas. The proportion of people from regional or remote areas was similar to the whole ASPREE cohort, despite the added difficulty of collecting Biobank specimens in these more remote locations.

Physical, lifestyle and clinical data closely resembled those of the ASPREE cohort at study enrolment (Tables 3 & 4). Although the ASPREE population can be considered initially healthy due to the inclusion/exclusion criteria of the clinical trial, Table 4 shows that many of the Biobank sub-group, like the ASPREE cohort in general, had medical or chronic conditions at baseline including diabetes mellitus (10%), hypertension (75%), previous cancer history (20%), low GFR (17%), osteoarthritis (36%) or indications of depression (9%). A large proportion of participants were taking prescription medicine (87%), including statins (38%).

**Table 3:** Physical and lifestyle measures at baseline by age group.

	Age range (yrs)				
	70–74	75–79	80–84	85+	Overall
	N = 7,314	N = 3,176	N = 1,342	N = 387	N = 12,219
Height (m, N = 12,204)	1.7 (0.1)	1.6 (0.1)	1.6 (0.1)	1.6 (0.1)	1.7 (0.1)
Weight (kg, N = 12,204)	78.6 (14.9)	76.2 (14.0)	73.4 (13.2)	69.8 (12.5)	77.1 (14.6)
Waist circumference (cm, N = 12,146)	97.5 (12.9)	97.0 (12.3)	96.3 (12.3)	94.7 (11.6)	97.1 (12.6)
BMI (kg/m^2^, N = 12,187)	28.3 (4.6)	28.0 (4.5)	27.3 (4.1)	26.5 (4.1)	28.0 (4.6)
BMI categories (kg/m^2^, N = 12 187)					
Underweight, <20	113 (2%)	45 (1%)	30 (2%)	12 (3%)	200 (2%)
Normal, 20–24.9	1,598 (22%)	760 (24%)	355 (27%)	131 (34%)	2,844 (23%)
Overweight, 25–29.9	3,271 (45%)	1,476 (47%)	658 (49%)	178 (46%)	5,583 (46%)
Obese, ≥30	2,308 (32%)	883 (28%)	295 (22%)	65 (17%)	3,551 (29%)
Current smoker	269 (4%)	88 (3%)	23 (2%)	10 (3%)	390 (3%)
Current Alcohol consumption	5,969 (82%)	2,449 (77%)	1,020 (76%)	295 (76%)	9,733 (80%)
Education (yrs)					
<12	3,450 (47%)	1,635 (51%)	687 (51%)	212 (55%)	5,984 (49%)
12–15	1,918 (26%)	834 (26%)	362 (27%)	103 (27%)	3,217 (26%)
16+	1,946 (27%)	707 (22%)	293 (22%)	72 (19%)	3,018 (25%)
Living alone	1,915 (26%)	1,093 (34%)	593 (44%)	216 (56%)	3,817 (31%)

Baseline characteristics are for those who contributed blood and/or urine to Biobank. Values are mean (SD) or proportion of column total (%). Participant numbers that differ from the overall N are stated within each variable category.

**Table 4:** Clinical measures at baseline by age group.

	70–74 Years	75–79 Years	80–84 Years	85+ Years	Overall
Hemoglobin (g/dL)	14.3 (1.2)	14.2 (1.2)	14.0 (1.2)	13.8 (1.2)	14.2 (1.2)
Fasting Glucose (mg/dL; n = 11 982)	98.8 (17.4)	99.0 (17.8)	98.2 (16.6)	98.3 (18.4)	98.7 (17.4)
HDL (mg/dL; n = 11 880)	61.1 (18.8)	61.1 (17.9)	62.6 (19.1)	63.5 (16.9)	61.3 (18.5)
Total Cholesterol (mg/dL; n = 12 077)	204.1 (37.6)	202.4 (37.8)	202.0 (37.9)	201.3 (39.2)	203.3 (37.8)
Serum/Plasma Creatinine (mg/dL; n = 11 874)	0.9 (0.2)	0.9 (0.2)	0.9 (0.2)	1.0 (0.3)	0.9 (0.2)
eGFR (ml/min/1.73m^2^; n = 11 874)	75.3 (13.2)	71.1 (13.4)	66.9 (13.6)	62.1 (14.2)	72.9 (13.8)
SBP (mmHg)	138.7 (15.9)	140.6 (16.3)	142.3 (16.7)	144.1 (16.2)	139.8 (16.2)
SBP≥160 mmHg	791 (11%)	414 (13%)	230 (17%)	77 (20%)	1,512 (12%)
DBP (mmHg)	78.0 (9.7)	76.7 (10.0)	75.2 (10.1)	74.5 (10.5)	77.2 (9.9)
DBP≥90 mmHg	907 (12%)	336 (11%)	120 (9%)	30 (8%)	1,393 (11%)
Heart rate (bpm)	71.1(10.5)	70.6 (10.7)	71.2 (10.7)	71.4 (11.5)	71.0 (10.6)
Diabetes mellitus^a^	681 (9%)	336 (11%)	137 (10%)	40 (10%)	1,194 (10%)
Hypertension	5,253 (72%)	2,456 (77%)	1,082 (81%)	321 (83%)	9,112 (75%)
Personal cancer history^a^	1,373 (19%)	643 (20%)	300 (22%)	98 (25%)	2,414 (20%)
eGFR<60 ml/min	942 (13%)	599 (19%)	409 (30%)	169 (44%)	2,119 (17%)
Osteoarthritis*^a^ (n = 5 677)	1,776 (24%)	843 (27%)	410 (31%)	122 (32%)	3,151 (26%)
Concomitant medication use^a^	6,267 (86%)	2,829 (89%)	1,201 (89%)	355 (92%)	10,652 (87%)
Statin use^a^	2,426 (39%)	1,094 (39%)	405 (34%)	112 (31%)	4,037 (38%)
Previous regular aspirin use	491 (7%)	260 (8%)	122 (9%)	41 (11%)	914 (7%)
CES-D-10 score >8	669 (9%)	318 (10%)	131 (10%)	37 (10%)	1,155 (9%)

Baseline characteristics (more details in [9]) data are for those who contributed blood and/or urine to Biobank. Total (Column N) is the same as for Table 2. CES-D-10 = Center for Epidemiologic Studies Short Depression Scale; DBP = diastolic blood pressure; eGFR = estimated glomerular filtration rate; HDL = high density lipoprotein; SBP = systolic blood pressure. Values are mean (SD or % of column total).

*A specific question about osteoarthritis was only asked after June 2013, hence not all participants were asked this question. BPs were the average of three readings taken with an automated monitor, 1 minute between readings. Heart rate is the average of three readings.

^a^ Self-report. Concomitant medication use refers to one or more prescription medications, not including study medication; Statin use = any statin. ^a^Definitions of chronic conditions: Diabetes mellitus = self-report of diabetes or fasting glucose ≥ 126 mg/dL or on treatment for diabetes; Hypertension = SBP ≥ 140 mmHg or DBP ≥ 90 mmHg or on treatment for high blood pressure.

### Clinical endpoints during the ASPREE clinical trial and early ASPREE-XT periods

Table 5 shows ASPREE major endpoints during the ASPREE clinical trial period (2010–2017, median follow-up of 4.7 years) and with the addition of a further 2.5 years of follow-up. Deaths and some of the major individual clinical events accrued by the Biobank cohort continued to rise over time, resulting in 11.2% who had died, 17.7% with cancer, 7.8% with cardiovascular disease events and 3.7% with physical disability by the nearly seven years of follow-up (all percentages of the original Biobank cohort). Investigators requiring access to ASPREE Biobank samples can request more detailed information about endpoints/cases including a breakdown of cancer and cardiovascular disease sub-types through the website, www.aspree.org.

**Table 5:** Clinical endpoints in ASPREE Biobank participants during ASPREE (2010–2017) and ASPREE+ASPREE-XT (2010–2020).

Clinical Event	During ASPREE		During ASPREE+ASPREE-XT	
	Participants with event + blood/urine (n, %)	Participants with event + DNA (n, %)	Participants with event + blood/urine (n, %)	Participants with event + DNA (n, %)
Death	573 (4.7%)	615 (4.5%)	1373 (11.2%)	1528 (11.2%)
Cancer*	1134 (9.3%)	1,259 (9.2%)	2161 (17.7%)	2408 (17.6%)
CVD*	564 (4.6%)	613 (4.5%)	953 (7.8%)	1060 (7.7%)
Dementia*	303 (2.5%)	332 (2.4%)	560 (4.6%)	619 (4.5%)
Major haemorrhage*	316 (2.6%)	357 (2.6%)	478 (3.9%)	539 (3.9%)
Persistent ADL loss**	228 (1.9%)	254 (1.9%)	456 (3.7%)	509 (3.7%)

Data are from the ASPREE-XT02 dataset for the period of 2010–2020 includes ASPREE (2010–2017) and ASPREE-XT (2018–2020). N = number of participants with event; % is proportion of the Biobank cohort contributing those samples. Biobank Cohort includes participants with any blood or urine (n = 12,219) or DNA (buffy coat or saliva, n = 13,684). DNA was from buffy coat or saliva. Clinical endpoints [9] were *adjudicated by expert panels after collection of documentation or **assessed by regular questionnaires. CVD is cardiovascular and cerebrovascular disease; ADL is severe difficulty completing or inability to perform independently for at least 6 months any one of six basic activities of daily living (walking across a room, toileting, bathing, eating, transferring from chair or bed, dressing).

## Discussion

The ASPREE Biobank was established to provide biospecimens from older adults prior to the diagnosis of major, life-limiting disease in order to track changes in biomarkers that may predict or diagnose early stage geriatric disease or inform clinical management. To this end, the methodological aims of the ASPREE Biobank for biospecimen collection, processing and storage were to: (a) ensure that all ASPREE participants regardless of geographical location or personal circumstances were provided the opportunity to donate biospecimens to the Biobank prior to initiation of randomised study medication or in the first year of the trial; (b) optimise the variety of blood components in different preservatives to provide samples for a wide range of future analyses; (c) minimise transport delays and maintain a cold processing environment between collection and storage for optimum quality samples; (d) provide availability of samples to many projects by fractionating into multiple aliquots in 2-D barcoded cryotubes, for durability in the very deep freeze; and (e) establish a safe, secure biorepository with accurate curation for practical retrieval of biospecimens for future projects.

All biospecimens are linked with extensive phenotypic data collected as part of the ASPREE trial including demographics, cognitive and physical function measures, blood pressure, health behaviours, basic and incidental activities of daily living, depression/mood, lifestyle, quality of life, prescription medications, clinical events, hospitalisations and frailty assessments [9]. Aliquots are curated in a manner that allows ready tracking and sample retrieval. The multiple low volume aliquots per participant minimises freeze-thaw cycles often associated when larger volume samples are retrieved from storage. Selection of sample types, collection and processing conditions and storage of aliquots were largely based on the UK Biobank’s sample collection protocols [12] which was the pre-eminent population-based biobank in 2008/9 when we were preparing the protocol for the ASPREE Biobank. The UK Biobank opted to ship samples overnight to a central laboratory for processing, which better suited their methods of set-up for collections from the hundreds of thousands of volunteers in the community. We opted for a shorter time between collection and storage, maintaining cold temperatures throughout handling, to preserve highly labile molecules. The selection of preservatives / additives was generally based on the UK Biobank’s protocol [12] to provide a wide selection of stored aliquots for multiple purposes.

ASPREE and the ASPREE Biobank have received many applications from investigators seeking to analyse specific biomarkers in ASPREE Biobank samples. These analyses range in focus from whole genome sequencing [13], targeted gene sequences [14], single nucleotide polymorphisms [15] and methylated DNA [16] to androgens measured by mass spectrophotometry [17], and to projects underway that include routine clinical chemistry, inflammatory and cardiovascular biomarkers, dementia biomarkers, lipidomics and proteomics. The types of samples most commonly requested from ASPREE Biobank have been buffy coat and saliva (providing DNA), serum and EDTA plasma. Since the requests for EDTA plasma and serum have been approximately equal in number, we are now collecting and storing equal numbers of these sample types in new waves of biobank collections. For genomics, the separation of the buffy coat sample provides high quality DNA for sequencing to elucidate gene-disease associations, potentially leading to development of new prevention strategies, identification of higher risk individuals for more intensive screening and early disease detection, and more effective treatments. Conversely, ‘gene-health’ associations can also be elucidated, providing insights into longevity and good health. Within the scope of cancer research, biobanked samples are vital for providing a comparator, or somatic genetic profile, to be referenced against the cancer tissue genetic profile [13].

A strength of the ASPREE Biobank is the broad geographical representation of older adults. The benefits of the introduction of tailor-made mobile laboratories (Biobuses) were evident in the numbers of participants recruited from regional and rural areas of Australia, providing compelling support for the principle of ‘taking science to the wider community’. Since many ASPREE participants lived outside the catchment of clinical trial centres or major hospitals, the Biobuses were the primary means of ensuring timely processing and very cold storage of samples from these participants based in regional areas. A further benefit of taking a mobile laboratory into regional communities was a raised awareness and interest in medical research. In regional areas and also in the city, the Biobuses were a drawcard at community venues where ASPREE study presentations to existing or potential participants were being held; they also provided media opportunities to promote the study and research in general.

The successful enrolment of ASPREE participants into the Biobank differed across the regions due to a number of factors including site remoteness, difficulties or delays in establishing a local Biobank laboratory and the strength of relationships built between the local ASPREE staff member and the ASPREE participants. Despite the tyranny of distance or other challenges, 75% of all ASPREE participants in Australia contributed samples of blood, urine or saliva for biobanking purposes. Furthermore, the strong links forged by ASPREE with general practitioners, the trust that developed between study participants and research staff, and regular communications with participants about the progress and findings of ASPREE, supported the retention and interest of participants in the sub-studies of ASPREE including the Biobank.

We found very few independent pathology services that were willing to accept research-based biobanking requests due to the specific requirements for sample handling, processing and storage which differ considerably from commercial pathology operations. On the other hand, pathology-based phlebotomy services near where the participant lived were convenient venues for sample collection and we had many successful arrangements with these agencies. In each case, we provided a courier staff member to attend the service at the time of the participant’s appointment to obtain written consent, if required, and to provide ice to transport the bloods back to our central laboratory, or Biobus, for processing and storage within 4 hours.

Few biobanks have targeted samples from older persons and most are disease focussed. Given the limited data available from older individuals for clinical or research-focussed biomarkers, the ASPREE samples may contribute to the development of ‘normal reference’ ranges for many analytes. Other large biobanks that enrolled community-based volunteers younger than ASPREE include the UK Biobank (40–69 years) [7] and the BWH/Harvard Cohorts Biorepository (a combination of biobanking activity from a number of studies including the Nurses’ Health Study, the Health Professionals Follow Up Study and Growing Up Today Study) (9–55 years) [6]. Due to its integration within the ASPREE trial, which was a ‘gold-standard’ clinical trial collecting detailed demographic, physiological and clinical data from each participant, the ASPREE Biobank has the opportunity to link with data from additional ancillary sub-studies, each with a focus on specific aspects of ageing health or disease. These studies involved brain and eye neuroimaging [18–20], hearing loss [21], sepsis [22], falls and fractures [23], obstructive sleep apnoea [20], and macular degeneration [24]. Results from all future biomarker analyses and genomic studies will be returned to the ASPREE database to further enrich the longitudinal clinical and phenotypic data that has now accrued for an average of more than 10 years through ASPREE and ASPREE-XT (www.aspree.org and [25]).

Adding value to the ASPREE Biobank is a further wave of blood and urine being collected during 2022–2024, extending the scope for investigations of ageing biomarkers, along with future follow-up of the cohort. This will enable further studies of biomarker trajectories and risk factors associated with ageing diseases.

## Conclusions

The ASPREE clinical trial provided the opportunity to establish the first of its kind: a healthy ageing Biobank. High quality physiological, demographic, cognitive, mood, physical function, quality of life and clinical data from the ASPREE trial and follow-on observational ASPREE-XT study are linked with the ASPREE Biobank specimens. The ASPREE Biobank is a population-based biobank conducted in a cohort of more than 12,000 initially healthy older people, making the samples useful resources for predicting future disease through biomarker analyses. The value of the specimen collection will increase as greater numbers of clinical outcomes accumulate, and more analytes are measured. As with other similar biospecimen repositories, ongoing support will be based on combinations of funding involving institutional support, cost recovery and ‘Research & Infrastructure’ grants.

## Supporting information

S1 Fig Timing of sample transport, processing & storage. Bar graphs show elapsed time for different stages of sample preparation, categorised into 30 min or 60 min blocks. (A) Sample transport time defined as the elapsed time from sample collection to sample arrival at the processing laboratory (n = 12,219). (B) Sample processing time defined as the elapsed time from sample arrival at the laboratory to storage in a freezer (n = 12,218). (C) Total time from collection to storage in a freezer (n = 12,218).
(DOCX)

S1 Table Biobank participant questionnaire data. Table reports the questions (abbreviated from actual) asked of participants at the time of biospecimen collections and the numbers (and percentage of total) of participants who recorded each answer. The options were “yes” or “no” or “unsure” with the latter numbers including those questions not answered. The differences in the questions at year 3 compared with baseline collections were mainly related to use of open-label aspirin and the ASPREE clinical trial medication.
(DOCX)
