# Supplementary material for: The ASPREE Healthy Ageing Biobank: Methodology and participant characteristics
Source: PLoS One. 2024 Feb 29;19(2):e0294743. doi: 10.1371/journal.pone.0294743 (PMC10903821; doi:10.1371/journal.pone.0294743)
Supplement: S1 Fig — Bar graphs show elapsed time for different stages of sample preparation, categorised into 30 min or 60 min blocks. (A) Sample transport time defined as the elapsed time from sample collection to sample arrival at the processing laboratory (n = 12,219). (B) Sample processing time defined as the elapsed time from sample arrival at the laboratory to storage in a freezer (n = 12,218). (C) Total time from collection to storage in a freezer (n = 12,218). (DOCX) [file pone.0294743.s001.docx]

***S1 Fig. Timing of sample transport, Processing & Storage***
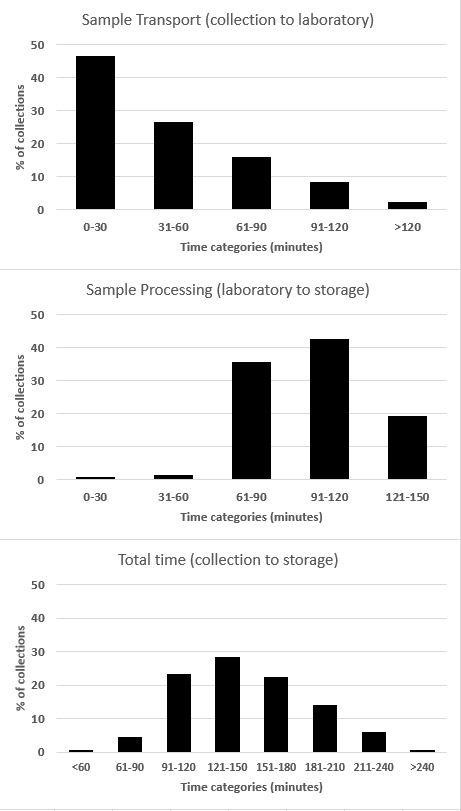


Bar graphs show elapsed time for different stages of sample preparation, categorised into 30 min or 60 min blocks. (A) Sample transport time defined as the elapsed time from sample collection to sample arrival at the processing laboratory *(n=12,219).* (B) Sample processing time defined as the elapsed time from sample arrival at the laboratory to storage in a freezer *(n=12,218).* (C) Total time from collection to storage in a freezer *(n=12,218).*
