# Supplementary material for: The ASPREE Healthy Ageing Biobank: Methodology and participant characteristics
Source: PLoS One. 2024 Feb 29;19(2):e0294743. doi: 10.1371/journal.pone.0294743 (PMC10903821; doi:10.1371/journal.pone.0294743)
Supplement: S1 Table — Table reports the questions (abbreviated from actual) asked of participants at the time of biospecimen collections and the numbers (and percentage of total) of participants who recorded each answer. The options were “yes” or “no” or “unsure” with the latter numbers including those questions not answered. The differences in the questions at year 3 compared with baseline collections were mainly related to use of open-label aspirin and the ASPREE clinical trial medication. (DOCX) [file pone.0294743.s002.docx]

***S1 Table. Biobank participant questionnaire data***

| **Baseline**  **(Number of participants and %)** | **Answer “Yes”** | **Answer “No”** | **Unsure or not answered** |  | **Year 3**  **(Number of participants and %)** | **Answer “Yes”** | **Answer “No”** | **Unsure or not answered** |
| --- | --- | --- | --- | --- | --- | --- | --- | --- |
| Last meal eaten >4hr ago? | 2085 (17.1%) | 10130 (82.9%) | 4  (0.0%) |  | Last meal eaten >4hr ago? | 1848 (17.4%) | 8760 (82.5%) | 9  (0.1%) |
| Has the participant started ASPREE trial medication? | 4167 (34.1%) | 8052 (65.9%) | 0  (0%) |  | Is the participant still taking ASPREE study medication? | 7257 (68.4%) | 3354 (31.6%) | 6  (0.1%) |
| Was aspirin taken in the last week? | 574 (4.7%) | 11645 (95.3%) | 0  (0%) |  | Was aspirin taken within the last week? | 887  (8.4%) | 9706 (91.4%) | 24  (0.2%) |
| Blood transfusion ever? | 1811 (14.8%) | 10133 (82.9%) | 275 (2.3%) |  | Blood transfusion in past year? | 1560 (14.7%) | 8564 (80.7%) | 493 (4.6%) |

Table reports the questions (abbreviated from actual) asked of participants at the time of biospecimen collections and the numbers (and percentage of total) of participants who recorded each answer. The options were “yes” or “no” or “unsure” with the latter numbers including those questions not answered. The differences in the questions at year 3 compared with baseline collections were mainly related to use of open-label aspirin and the ASPREE clinical trial medication.
